# Smith–Lemli–Opitz Syndrome: Oral Characteristics and Risk Factors for Caries Development

**DOI:** 10.3390/biomedicines13030574

**Published:** 2025-02-25

**Authors:** Dorota Olczak-Kowalczyk, Aneta Witt-Porczyk, Paula Piekoszewska-Ziętek, Małgorzata Krajewska-Walasek

**Affiliations:** 1 Department of Paediatric Dentistry, Medical University of Warsaw, St. Binieckiego 6, 02-097 Warsaw, Poland; 2 Private Dental Practice, Warsaw, Poland; aneta_witt@o2.pl; 3 Department of Medical Genetics, Children’s Memorial Health Institute, St. Dzieci Polskich 20, 04-736 Warsaw, Poland; m.walasek@czd.pl

**Keywords:** dental caries, genetic diseases, medical genetics, oral diagnosis, oral medicine, oral pathology

## Abstract

**Background/Objectives**: Smith–Lemli–Opitz syndrome is a metabolic autosomal recessive disease, characterized by congenital defects, with concomitant psychomotor developmental delay. The symptoms are variable and depend on the clinical form of the disease. The aim of this study was to assess the prevalence and types of oral abnormalities in children and adolescents with Smith–Lemli–Opitz syndrome. **Methods**: The study enrolled 30 patients, including 15 subjects with Smith–Lemli–Opitz syndrome, confirmed by a genetic examination. We performed an extra- and intraoral examination, paying attention to the presence of dysmorphic features, including the shape and symmetry of the face; the condition of the skin and lips; the gingival and hygienic status; dental caries; lesions of non-carious origin; abnormalities of size, shape, and number; and alignment of teeth in the dental arch. **Results**: Patients in the study group presented with micrognathia, a short neck, ptosis, and an upturned nose. More frequently than in the control group, we observed occlusal abnormalities and dental crowding, anatomical abnormalities or gingivitis. The prevalence of dental caries in both groups was similar; however, the study group recorded higher dmft and DMFT values. **Conclusions**: The phenotypic features of patients with Smith–Lemli–Opitz syndrome increase their risk of developing dental caries and gingivitis.

## 1. Introduction

Smith–Lemli–Opitz syndrome (SLOS) is a metabolic autosomal recessive disease [1]. It is relatively uncommon in Europe. The incidence of SLOS is estimated at 1/10,000 to 1/70,000 in central and northern Europe and 1/50,000 in the US. However, the incidence is probably higher than these estimates due to undiagnosed mild forms of the disease and fetal death in utero for severe and fatal forms [2]. The numerous defects in patients with this syndrome are connected with the absence or reduced activity of 7-dehydrocholesterol reductase (DHCR7), caused by a mutation of the DHCR7 gene encoding the reductase protein, which is located on chromosome 11 in the q12–q13 region [3,4]. Expression of this gene occurs in the adrenal glands, liver, small intestine, and brain. DHCR7 catalyzes the final step in cholesterol biosynthesis [3]. Smith–Lemli–Opitz syndrome is diagnosed in individuals presenting with characteristic clinical features, along with elevated levels of 7-dehydrocholesterol, and/or the identification of biallelic pathogenic or likely pathogenic variants in the DHCR7 gene, through molecular genetic testing. Low cholesterol levels cause a wide spectrum of clinical symptoms, which range from mild to fatal [5].

SLOS is characterized by congenital defects, with concomitant psychomotor developmental delay. In the neonatal period, variable muscle tone is noted: initially, flaccidity, then increased muscle tone, and gastrointestinal disorders (vomiting, diarrhea, constipation [5]). Permanent symptoms include growth disorders, poor weight gain, and intellectual disability. In addition, patients may have behavioral disorders with autistic features, as well as a specific facial and head appearance [6,7]. Patients are observed to have wide-set eyes; drooping eyelids; a short, upturned nose, with a broad ridge; low-set, often large ears, with a protruding lobe and thin rim; and a small mandible [1,8]. There may be abnormalities in the structure of many organs, such as defects of the lower and upper limbs, defects of the heart and kidneys, defects of the gastrointestinal tract, and tactile hypersensitivity of the feet, hands, and face [5,7,8]. The occurrence of individual symptoms is variable and depends on the clinical form of the disease.

In mild forms, one observes, among other things, discrete features of facial dysmorphia, syndactyly of the second and third toes, eating disorders, moderate delays in psychomotor development, difficulties at school, and behavioral disorders. In classical and severe forms, a child with SLOS is born with numerous congenital defects and strongly marked features of facial dysmorphia. There are malformations of the heart, gastrointestinal tract, abnormalities of the external genitalia, microcephaly, decreased muscle tone, delayed psychomotor development, psychomotor hyperactivity, aggression, autistic features, and syndactyly of the second and third toes [1,5,8]. Some patients with SLOS show increased susceptibility to infections, including respiratory, ear, and urinary tract infections [5]. Delayed psychomotor development, dental abnormalities, and occlusal abnormalities, as well as impaired homeostasis of the oral environment, due to frequent vomiting, may increase the risk of dental caries and gingivitis in children with Smith–Lemli–Opitz syndrome [9,10,11]. Some symptoms of the syndrome can be alleviated with cholesterol and bile acid supplementation, and the use of statins, such as simvastatin, to lower 7-DHC levels. Oral health symptoms can be reduced with properly targeted preventive and dental treatment measures [10]. However, early recognition of the disease is important, and GPs, including dentists, should be involved in this process. It is essential to know the symptoms indicative of SLOS, as well as the oral health risks and causative factors of tooth decay and gingivitis in this group of patients.

Dental caries is primarily caused by the demineralization of tooth enamel due to acids produced by bacteria, such as Streptococcus mutans, metabolizing fermentable carbohydrates. In SLOS, abnormalities in cholesterol metabolism can lead to various systemic and oral manifestations. While there is no direct evidence linking cholesterol metabolism defects in SLOS to an increased risk of dental caries, the syndrome’s associated oral anomalies, such as enamel hypoplasia, delayed tooth eruption, and malocclusion, may create environments conducive to bacterial colonization and plaque accumulation, thereby elevating the risk of cavities. Additionally, feeding difficulties and dietary modifications common in SLOS patients could influence oral bacterial profiles and caries risk [12].

Individuals with Smith–Lemli–Opitz syndrome often face challenges in accessing appropriate dental care due to the rarity and complexity of their condition. Specialized dental care is essential for children with genetic syndromes, to address their unique oral health needs. Our recent study, conducted in 2024, revealed that most children with genetic diseases, including SLOS, benefited from dental care, provided as a result of insurance coverage, at specialized centers [13]. However, specific data regarding the insurance status (public or private) of SLOS patients and its impact on dental care access are limited. Currently, there are no standardized, evidence-based guidelines specifically for the dental management of SLOS patients. However, due to the complex medical and behavioral characteristics of SLOS, it is recommended that these individuals receive comprehensive, multidisciplinary care.

The aim of this study was to assess the type and prevalence of oral abnormalities in children and adolescents with Smith–Lemli–Opitz syndrome, including gingivitis and dental caries, to assess their oral behaviors, and to identify factors associated with the development of dental caries in SLOS patients.

## 2. Materials and Methods

The project received ethical approval from the relevant Bioethics Committee (KB/228/2009 approval number) and was conducted in accordance with the Declaration of Helsinki. Informed written consent was collected from the patient and/or his/her guardians prior to their participation in the research.

The study enrolled 30 patients (17 girls and 13 boys), aged between 3 and 17.8 years, including 15 subjects with SLOS (mean age 6.66 years ± 4.13), confirmed by a genetic examination, and 15 generally healthy subjects (mean age 6.66 years ± 3.60). The subjects were recruited from among the patients at the Department of Medical Genetics and the Department of Pediatric Dentistry. Inclusion in the study group required a diagnosis of Smith–Lemli–Opitz syndrome, confirmed by a geneticist. Generally healthy patients undergoing treatment at the Department of Pediatric Dentistry were randomly recruited for the control group. The age range of the patients in the control group was similar to that of the SLOS patients. Exclusion criteria for the control group were a history of chronic general illness or chronic medication use. Also, patients with developmental abnormalities of dentition or the oral cavity were excluded from the study.

Table 1 presents the characteristics of the study and control groups by gender, age, and type of dentition.

An interview and a physical examination were conducted. The assessment of the patient’s history included information on general diseases and medications taken, and their hygienic and dietary behavior regarding their oral health (frequency of tooth brushing, use of floss, mouthwash, frequency of consumption of cariogenic products). The questionnaire used to assess the hygiene and dietary behavior of the patient was developed by our research team for the specific purpose of this study. The questionnaire was designed to gather relevant information. While it is not a standardized or previously validated tool, the questionnaire was structured based on established principles of dietary and oral hygiene assessments. The physical examination was conducted by a single examiner, according to the principles of dental and orthodontic diagnosis. The clinical examination included an extraoral and intraoral examination. In the extraoral part, attention was paid to the presence of dysmorphic features that are characteristic of SLOS, including the shape and symmetry of the face, and the condition of the skin, lips, and corners of the mouth. During the intraoral examination, the patient’s gingival status was assessed, including the presence and type of morphological abnormalities and inflammation, using the Gingival Index (GI) by Löe and Sillness. The Plaque Index (PI), according to Löe and Sillness, was used to assess the patient’s oral hygiene status. In this study, we categorized oral hygiene based on the Plaque Index (PI). The classification was as follows: scores 0–1.0 for good oral hygiene; 1.1–2.0 for average oral hygiene; and 2.1–3.0 for poor oral hygiene. This categorization follows established standards in dental research and allows for a clear assessment of oral hygiene status among the study participants [14]. In assessing the condition of the teeth, attention was paid to the presence of carious cavities (ICDAS-II); lesions of non-carious origin (modified DDE index); abnormalities of size, shape, and number; and alignment of teeth in the dental arch. The prevalence of caries was determined (dmft/DMFT). Due to the inability to perform a radiographic diagnosis in regard to most SLOS patients, dental hypodontia was diagnosed in the case of missing teeth that were not caused by local causes (exfoliation, extraction, eruption). The occlusal conditions were analyzed. During the evaluation of oral anatomy, attention was paid to the shape of the alveolar processes, the palatal arches, the size of the tongue, and the tension of the orbicularis oris muscle.

CRT Bacteria commercial tests (Ivoclar Vivadent) were used for the bacteriological evaluation. The quantity of Streptococcus mutans and Lactobacillus spp. in stimulated saliva was determined after 48 h of incubation at 37 °C, based on a macroscopic evaluation of the number of colonies on the medium, by comparing the sample with the graphically presented standard results included in the kit. The test was performed, according to the manufacturer’s recommendations, in the morning, at least 2 h after the last oral meal and dental cleaning procedure.

The results obtained were subjected to statistical analysis. An evaluation of the significance of the differences between the genetic syndrome group and the control group in regard to the quantitative traits was carried out using Student’s t-test. An evaluation of the interaction between the parameters considered was carried out using Spearman’s rank correlation analysis. A significance level of *p* > 0.05 was adopted.

This study complies with the STROBE guidelines.

## 3. Results

The findings on the hygienic and dietary behaviors of the patients in both study groups are shown in Table 2. Dental care was received by eight (53.33%) of the SLOS patients. Only one child in the SLOS group received orthodontic treatment, despite the presence of malocclusion in all subjects (Table 3).

**Figure 1:**
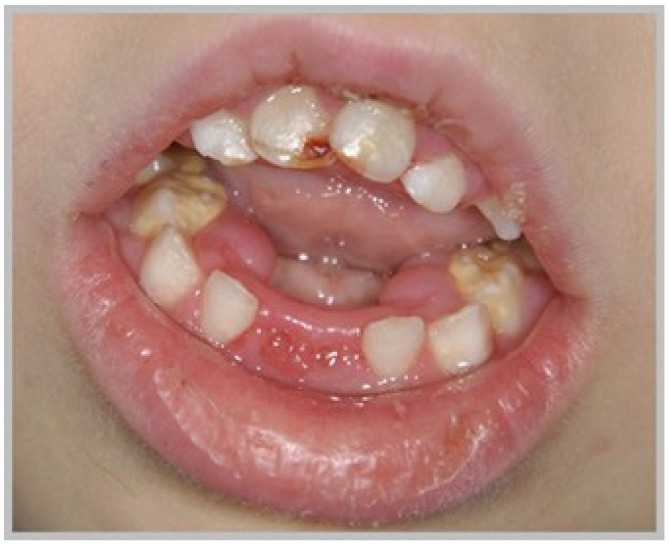
Hypotonia of the orbicularis oris muscle; dryness of the lips; thickened, irregularly shaped alveolar part of the mandible; calculus deposits; and gingivitis in a 5.5-year-old boy with SLOS.

As identified during the extraoral examination, all patients with SLOS had a small head, micrognathia, a short neck, ptosis, and an upturned nose. More frequently than in the control group, we observed occlusal abnormalities and dental crowding, and anatomical abnormalities in the oral cavity, such as a cleft palate (26.66%), gothic palate (73.33%), and hypotonia of the orbicularis oris muscle (66.66%). In the SLOS patients, we noticed Class II malocclusions (93.33%) and the crowding of teeth (86.66%). Only in patients with SLOS was the alveolar process collared (Table 3, Figure 1). Only in the SLOS group were enamel defects noted in regard to both deciduous and permanent dentition. In regard to the deciduous dentition, these lesions were enamel opacities, accompanied by hypoplasia (*n* = 2; 13.33%). In regard to the mixed dentition, one (6.66%) of the subjects showed the presence of opacities in the enamel of deciduous teeth and opacities and hypoplasia in the enamel of permanent teeth. In regard to the permanent dentition, one (6.66%) of the study subjects had opacities and enamel hypoplasia.

One SLOS patient with permanent dentition had abnormalities regarding the number of teeth, i.e., missing tooth germs, namely 12 and 22, which was confirmed radiographically. One boy presented with a double tooth, 52.

Participants with SLOS were diagnosed with gingivitis more often than the patients in the control group, and the condition was more severe (GI 0.83 ± 0.84 and 0.08 ± 0.20, respectively; *p* = 0.002) (Table 3). No gingival recession or proliferative lesions were found in any of the study subjects.

Hygienic negligence was found in 60% of subjects with Smith–Lemli–Opitz syndrome, which included five subjects with an unsatisfactory hygiene status (33.33%). The mean value of the PI in this group was significantly higher than in the control group (1.74 ± 0.84 vs. 1.04 ± 0.37; =0.006).

The prevalence of dental caries in both groups was similar; however, the group with SLOS recorded higher dmft and DMFT values. Spearman’s correlation analysis between dental caries, health behaviors, and oral abnormalities, such as malocclusion, enamel malformation, GI, and PI values, in the SLOS group, showed a statistically significant relationship with dietary behavior, hygiene, and gingival status only (Table 4). In the control group, the relationship between dmft and teeth brushing twice a day was significant. In this group, there were no other significant associations between health behaviors and oral health parameters.

## 4. Discussion

Smith–Lemli–Opitz syndrome is one of the rare genetic diseases. In the literature, only a few publications describe the characteristic features observed in the oral cavity of these patients, based on the description of single cases [15,16,17,18]. All authors point out characteristic dysmorphic features in the orofacial region and the presence of hypotonia of the orbicularis oris muscle and mouth breathing. Among the anatomical abnormalities, researchers describe the presence of a gothic palate [10,17], macroglossia [10], a cleft palate [15,16], wide alveolar processes [15], and dental abnormalities, such as premature eruption of deciduous teeth [15], enamel developmental defects [15], fusion of deciduous teeth [12], the presence of supernumerary deciduous teeth [15], and hypodontia [10]. Teeth crowding [16,17], retrognathism [10], and an open bite [15] are frequently observed, as well as hygienic neglect, generalized gingivitis, and advanced tooth decay [10,15,17]. Su-Hyun [18] described a case of intranasal teeth that were associated with a cleft lip and alveolar processes in a patient with SLOS.

In the group of 15 patients with Smith–Lemli–Opitz syndrome, all of them had malocclusions and wide, collared alveolar processes. According to the literature, a cleft palate occurs in 40–50% of patients with SLOS [16,19,20]. In the present study, this defect was reported in 26.6% of the subjects, which is similar to the results obtained by Jezela-Stanek et al. [11]. Discussions have taken place about whether these malformations are the result of low cholesterol or the buildup of sterol precursors. According to Engelking et al. [21], sterol precursor accumulation plays a fundamental role in the genesis of facial malformations like cleft palate.

Other oral abnormalities were present only in some of the subjects. A limitation of this study is certainly the lack of a radiographic diagnosis in regard to all the subjects, which makes it impossible to determine the frequency of missing teeth or the presence of supernumerary teeth in the entire study group. Rojare et al. [10] presented a description of two patients with SLOS who were not evaluated for oral anomalies until they were 14 and 15 years old. Both patients were clinically diagnosed with dental agenesis.

Similar to the observations by other researchers, we noted a significantly worse oral hygiene status in SLOS patients compared to the controls, and a statistically significant higher incidence of gingivitis in this group of patients. A predisposition to gingivitis in SLOS patients has also been reported by other researchers [15,17]. Rojare et al. [10] diagnosed gingivitis and gingival hypertrophy in a 15-year-old boy, while Diaconescu et al. [17] presented a case of a teenage girl with chronic generalized gingivitis and gingival hyperplasia. However, in both studies, there is no information on their oral hygiene status. It is noteworthy that some patients show greater susceptibility to infection, which may manifest as a predisposition to gingivitis.

In the group we surveyed, most caregivers of SLOS patients admitted that they brush their child’s teeth only once a day. Certainly, performing effective hygiene procedures is difficult for caregivers because of the child’s mental disabilities, motor disorders, and severe oral hypersensitivity. This condition requires caregivers to be extremely patient and motivated to perform oral hygiene procedures for the children in their care. In addition, the presence of lip and tongue dysfunction and hypotonia of the orbicularis oris muscle, as well as the presence of wide, distended alveolar processes, malocclusion, and crowding of the teeth, hinder natural cleaning of the oral cavity and promote the accumulation of bacterial plaque [9,10,11]. These aspects are also pointed out by Rojare et al. [10] and Matthews-Brzozowski et al. [12]. Matthews-Brzozowski et al. [12] documented a case of a boy suffering from SLOS, who had his teeth brushed by his parents and who had also been taught to use the toothbrush by himself, which demonstrates that involvement of the patient can lead to habituation in regard to oral care procedures. However, the patient’s oral hypersensitivity was significant enough that the boy did not allow an intraoral examination to be performed by a dentist.

The nutritional disorders present in SLOS subjects, which are related to a dietary regimen of frequent, low-volume meals, and frequent vomiting, are additional factors contributing to the disruption of the oral ecosystem that promotes caries [7]. In the present study, the level of caries in both deciduous and permanent teeth was higher among subjects with SLOS than in the control group. In addition to the relationship between caries and oral hygiene, caries was also shown, in this group, to be associated with the frequent consumption of sweets and giving the child more than three snacks. It is noteworthy that dietary behavior regarding the frequency of cariogenic products was significantly worse in the control group. Nevertheless, there was no strong relationship between diet and caries in the control group, which may be related to more frequent hygiene procedures being carried out by this group and their effectiveness.

Nutritional abnormalities present in SLOS patients may also be the cause of abnormal structures and abnormalities in the formation of protein structures and the mineralization of hard dental tissues. In the SLOS group we studied, these abnormalities were observed in both deciduous (25%) and permanent teeth (33.33%). They had the complex character because hypoplasia was accompanied by tooth enamel opacities. These results coincide with those obtained by Pizzo et al. [15]. Developmental defects in the enamel may increase the susceptibility of teeth to acids, which may hinder the cleaning of the teeth, and, thus, increase the risk of dental caries in individuals with SLOS. Our statistical analysis, however, did not show an association with dental caries, which is probably related to the small number of patients with enamel defects. Massignan et al. [22] stated that the likelihood of dental caries is proven to be more than three times higher in teeth with underdeveloped enamel compared to teeth without enamel defects (OR = 3.10; 95% CI: 1.91; 5.01).

Children with SLOS often arrive at dental offices too late, as evidenced by the large disparity between the number of teeth with decay (dt/DT) and the number of teeth with fillings (ft/Ft) and the lack of orthodontic treatment. Doctors caring for SLOS patients point out that it is necessary to carry out a dental diagnosis and start treatment as early as possible. Cooperation with the parents of the children is key. Positive results in regard to either prevention or dental treatment depend specifically on the doctor’s cooperation with the child’s parents [12]. The authors of studies focusing on SLOS underline that preventive dental procedures can be performed with respect to children suffering from this condition [15]. Orthodontic treatment can be challenging. Muzzin et al. [16] reported that an attempt to treat an adult patient with SLOS (diagnosed with Class II malocclusion and crowded teeth) was unsuccessful due to the patient’s failure to accept the orthodontic appliance in their mouth.

This study has several limitations. First, its observational nature prevents the establishment of causal relationships, making the findings primarily descriptive. All the participants were recruited from a single clinical setting, which may introduce bias and limit the generalizability of the results. The control group in this study was composed of generally healthy children, recruited from the pediatric dentistry clinic at our institution. As an academic medical center, our clinic serves a diverse population, including both patients who attend regular dental visits and those who seek care sporadically or for specific treatment needs. The selection of the control group participants was conducted randomly from among the children who met the inclusion criteria, without a specific focus on those undergoing regular dental check-ups. This approach ensured that the control group represented a broad spectrum of oral health conditions, rather than being limited to individuals receiving continuous preventive care. While we acknowledge that recruiting controls from the same institution as the study group may introduce potential bias, this methodology allowed standardized clinical assessments to be carried out, ensuring consistency in regard to the examination procedures and diagnostic criteria. Nevertheless, we recognize this as a limitation, acknowledge that the measurements should be interpreted with caution, and emphasize the need for future studies to explore control groups from varied settings to further validate the findings. Additionally, not all potential biases were considered, such as differences in access to healthcare, socioeconomic status, and environmental factors. This study did not document certain variables, like the patient’s history of increased vomiting and its impact on dental erosion, which could have provided additional insights. There is also the possibility that confounding factors influenced the results, which should be taken into account when interpreting the findings.

In conclusion, the phenotypic features of SLOS patients, particularly intellectual disability, collared alveolar processes, malocclusion, and crowded teeth, contribute to plaque accumulation and hinder effective oral hygiene. Children with SLOS had a higher risk of caries and gingivitis, compared to the control group. Dental negligence in terms of prevention and treatment is the reason for tooth extraction and the deterioration of children’s quality of life, so it is essential to include SLOS patients in regular dental care. It is necessary to emphasize the need for the practical education of parents and caregivers on the frequency and technique of performing hygienic procedures in regard to children, taking into account morphological anomalies. Patients with Smith–Lemli–Opitz syndrome require comprehensive and individualized dental care due to their increased risk of experiencing oral health issues, including dental caries, gingivitis, enamel defects, and malocclusions. Given the challenges associated with this condition, such as intellectual disability, motor impairments, and oral hypersensitivity, routine dental care should be adapted to their specific needs. This can include early preventive measures, modified oral hygiene approaches (caregivers should receive guidance on specialized toothbrushes or adapted brushing techniques), and enhanced caries prevention strategies (individualized prophylactic strategies). Dental care should be integrated into their primary healthcare plan, with close coordination between pediatricians, geneticists, and dentists to monitor systemic health factors that may influence oral health.

Future research on Smith–Lemli–Opitz syndrome should focus on larger multicenter studies to improve sample diversity and reduce selection bias. Longitudinal follow-up studies would provide insights into the progression of oral health issues and the effectiveness of preventive measures. Incorporating socioeconomic factors and using advanced diagnostic tools, such as salivary biomarkers or microbiome analysis, could enhance the understanding of caries risk in this population. Finally, integrating interdisciplinary care and comparing SLOS patients with other genetic syndromes could lead to more tailored and comprehensive treatment recommendations.

## Figures and Tables

**Table 1:** Characteristics of the study and control group.

Group	Total *n*/%	Gender		Age (Years) Mean ± SD	Dentition		
		F, *n*/%	M, *n*/%		Primary *n*/%	Mixed *n*/%	Permanent *n*/%
SLOS	15/100.00	7/46.66	8/53.33	6.66 ± 4.13	9/60.00	3/20.00	3/20.00
Control	15/100.00	10/66.66	5/33.33	6.66 ± 3.60	9/60.00	3/20.00	3/20.00

**Table 2:** Hygienic and dietary behaviors of the patients in the study groups.

Behavior		SLOS *n* = 15 (100%)	Control *n* = 15 (100%)	*p*
		*n* (%)		
Using mouthrinse		0/15 (0.00)	0/15 (0.00)	1.000
Using dental floss		0/15 (0.00)	3/15 (20.00)	0.223
Teeth brushing	No brushing	1/15 (6.66)	0/15 (0.00)	1.000
	1 per day	10/15 (66.66)	0/15 (0.00)	0.000 *
	2 per day	4/15 (26.66)	15/15 (100.00)	0.000 *
>3 sweet snacks per day		3/15 (20.00)	13/15 (86.66)	0.004 *
Sticky foods	≥1 per day	2/15 (13.33)	7/15 (46.66)	0.111
	2–3 times in a week	1/15 (6.66)	5/15 (33.33)	0.170
	Once a week	12/15 (80.00)	3/15 (20.00)	0.003 *
Sweets	≥1 per day	5/15 (33.33)	13/15 (86.66)	0.009 *
	2–3 times in a week	2/15 (13.33)	2/15 (13.33)	1.000
	Once a week	8/15 (53.33	0/15 (0.00)	0.003 *
Sweet drinks	≥1 per day	7/15 (46.66)	13/15 (86.66)	0.052
	2–3 times in a week	1/15 (6.66)	3/15 (20.00)	0.591
	Once a week	7/15 (46.66)	0/15 (0.00)	0.009 *

* Statistically significant.

**Table 3:** Oral health status and salivary levels of *S. mutans* and *Lactobacillus* spp. bacteria in subjects with Smith–Lemli–Opitz syndrome and the controls.

Parameter		SLOS *n* = 15 (100%)	Control *n* = 15 (100%)	*p*
		*n* (%)		
Anatomical abnormalities of the oral cavity	Collared alveolar process	15/15 (100)	0/15 (0.00)	<0.00001 *
	Gothic palate	11/15 (73.33)	2/15 (13.33)	0.003 *
	Cleft palate	4/15 (26.66)	0/15 (0.00)	0.107
Hypotonia of the orbicularis oris muscle		10/15 (66.66)	2/15 (13.33)	0.009 *
Malocclusion	Overall	15/15 (100)	5/15 (33.33)	0.009 *
	Class II	14/15 (93.33)	3/15 (20.00)	0.002 *
	Crossbite	0/15 (0.00)	2/15 (13.33)	0.464
	Deep bite	1/15 (6.66)	0/15 (0.00)	1.000
	Class III	0/15 (0.00)	1/15 (6.66)	1.000
	Open bite	0/15 (0.00)	0/15 (0.00)	1.000
Developmental defects of the enamel	Primary teeth	3/12 (25.00)	0/12 (0.00)	1.000
	Permanent teeth	2/6 (33.33)	0/6 (0.00)	0.441
State of gingiva	GI ≥ 0.1	10/15 (66.66)	3/15 (20.00)	0.027 *
Oral hygiene	PL I ≥ 1.1	9/15 (60.00)	3/15 (20.00)	0.062
Teeth crowding		13/15 (86.66)	3/15 (20.00)	0.006 *
		Mean ± SD		
Caries	DMFT	6.00 ± 7.92	2.16 ± 2.04	0.000 *
	dmft	3.91 ± 5.97	2.91 ± 3.17	0.844
		*n* (%)		-
	dmft/DMFT > 0	9 (60.00)	8 (53.33)	0.981
Lactobacillus spp.	>10^5^ CFU/mL	3 (20.00)	1 (6.66)	0.591
*Streptococcus mutans*	>10^5^ CFU/mL	3 (20.00)	1 (6.66)	0.591

GI—Gingival Index; PL I—Plaque Index; * statistically significant.

**Table 4:** Spearman’s correlation coefficients related to statistically significant health behaviors and oral health parameters associated with dental caries in SLOS group.

Parameter	dmft/DMFT > 1	dmft	DMFT
	r		
>3 sweet snacks	0.520 *	0.327	0.165
Frequency of sweet intake	0.772 *	0.306	0.540 *
Plaque Index	0.681 *	0.137	0.640 *
Gingival Index	0.803 *	0.474	0.447

* Statistically significant *p* < 0.05.

## Data Availability

The data that support the findings of this study are not openly available due to reasons of sensitivity and are available from the corresponding author upon reasonable request. The data are located in controlled access data storage at the Medical University of Warsaw.
